# Commissioning and Validation of a Synchrocyclotron – Based Ultra-High-Dose-Rate Beamline for FLASH

**DOI:** 10.1016/j.ijpt.2026.101323

**Published:** 2026-06-11

**Authors:** Yuting Lin, Jufri Setianegara, Aoxiang Wang, Nicolas Gerard, Jarrick Nys, Rudi Labarbe, Erik Traneus, Hao Gao, Ronald C. Chen

**Affiliations:** 1 Department of Radiation Oncology, University of Kansas Medical Center, Kansas City, KS, USA; 2 Ion Beam Applications (IBA), Louvain-la-Neuve, Belgium; 3 RaySearch Laboratories AB, Stockholm, Sweden; 4 Department of Biomedical Engineering, Huazhong University of Science and Technology, Wuhan, China; 5 Department of Radiation Oncology, UT Southwestern Medical Center, Dallas, TX, USA; 6 Department of Radiation Oncology, University of Pennsylvania, Philadelphia, PA, USA

**Keywords:** Proton therapy, Ultra-high dose rate (UHDR), FLASH, Synchrocyclotron, Pencil beam scanning (PBS)

## Abstract

**Purpose:**

FLASH radiotherapy delivers ultra-high dose rate radiation (>40 Gy/s) has shown promise in reducing normal tissue toxicity while maintaining tumor control. IBA’s single-room proton system, equipped with S2C2 superconducting synchrocyclotron accelerator, has recently demonstrated to achieve UHDR delivery. Integrating the UHDR beam line in the treatment planning system (TPS) is crucial for accurate dose calculation in preclinical study, optimization of the 2D dose profile as well as paving the way for further accessory development for spread-out Bragg peak FLASH. This study aims to commission and validate a synchrocyclotron-based pencil beam scanning UHDR proton beamline on the IBA ProteusONE system in RayStation TPS. The goal is to establish a framework for TPS modeling and validation, facilitating preclinical FLASH radiotherapy studies.

**Methods:**

The transmission UHDR beamline using scanning proton beam energy of 228 MeV was characterized at gantry 0° using comprehensive point dose and 2D lateral profiles measurements. The beam model was developed in RayStation, incorporating key parameters such as virtual source position, spot size, integrated depth dose (IDD), and absolute dose calibration. Extensive validations were conducted using ionization chambers, film dosimetry, and 2D scintillation detectors, with gamma analysis performed to assess the accuracy of the TPS model with open field and field in the presence of brass apertures.

**Results:**

The UHDR beamline achieved ultra-high dose rates exceeding 40 Gy/s (average dose rate for a 2.5 x 2.5 cm field) with consistent dose output validated across multiple detectors. The nozzle current was measured to be linear with respect to the requested MU in the range of 45 to 126 nA. The RayStation beam model demonstrated excellent agreement with experimental measurements, achieving less than 2.5% deviation for all point dose measurements. For 2D profile measurements, gamma passing rates >95% under 2%/2 mm criteria for all fields. The TPS allowed optimization of the spot pattern for UHDR FLASH beams aligned closely with clinical beam profiles, enabling accurate preclinical study comparisons.

**Conclusions:**

A synchrocyclotron-based UHDR beamline was successfully commissioned and validated through a reliable TPS model for transmission FLASH application. The results provide a foundation for preclinical FLASH-RT research and future clinical applications, demonstrating the feasibility of integrating FLASH-RT into existing proton therapy platforms. Future work will extend the commissioning to all gantry angles and explore spread-out Bragg peak FLASH delivery for improved dose conformality.

## Introduction

FLASH radiotherapy (FLASH-RT), defined as ultra-high dose rate (UHDR) radiotherapy, has gained significant attention in recent years 1, 2, 3, 4, 5, 6, 7 Typically, FLASH-RT involves delivering dose rates exceeding 40 Gy per second. Numerous preclinical studies have demonstrated that irradiation at FLASH dose rates provides superior normal tissue protection compared with conventional dose rates, leading to a distinct radiobiological phenomenon known as the *FLASH effect*.^8^, ^9^, ^10^, ^11^, ^12^, ^13^ This effect is generally observed in normal tissues but not in tumor tissues, suggesting that FLASH irradiation can selectively spare healthy tissue from radiation-induced injury while maintaining equivalent tumor control. As a result, FLASH-RT represents a promising approach for improving the therapeutic ratio in radiotherapy.^9^, ^10^, ^14^, ^15^ Several radiation modalities have been explored for implementing FLASH-RT, including photons, electrons, protons, and some heavier particle beams.^16^, ^17^, ^18^, ^19^, ^20^, ^21^ Among these, protons have emerged as a particularly promising option for broad clinical translation, as they can achieve UHDRs for deep-seated targets while minimizing the exit dose.^2^, ^6^ Furthermore, proton FLASH delivery can be realized using existing clinical accelerator infrastructure with appropriate beam modifications. Recent feasibility studies have demonstrated the safe and effective delivery of proton FLASH-RT in early-phase clinical trials involving human patients.^18^, ^22^, ^23^

Among the proton delivery technologies capable of FLASH dose rates, synchrocyclotron-based proton pencil beam scanning (PBS) systems—widely employed in single-room proton therapy facilities—have emerged as an important platform for implementing FLASH-RT.^6^, ^24^ The University of Kansas Cancer Center operates a single-room proton therapy system equipped with IBA’s S2C2 superconducting synchrocyclotron. In this system, UHDRs have been demonstrated by increasing the proton charge per pulse while maintaining a constant pulse width and optimizing beamline transmission efficiency.^25^, ^26^, ^27^, ^28^, ^29^

Re-optimization of the beamline parameters to achieve UHDRs resulted in beam energies and spot profiles that differ from those of the standard clinical beam at its maximum energy. This study focuses on the commissioning and validation of the UHDR beamline for the IBA ProteusONE system. The primary objective is to establish a robust and reproducible framework for implementing FLASH-RT within the RayStation treatment planning system (TPS) environment. The work consists of 3 main components.

First, a comprehensive dosimetric characterization and TPS modeling of the UHDR beamline were performed, establishing a methodology that can be applied to other synchrocyclotron-based systems with similar configurations. Second, validation measurements were conducted at a gantry angle of 0°, including absolute dose measurements with multiple detectors and dose profile comparisons to ensure agreement between modeled and measured data. Finally, the benefit of incorporating the FLASH model into RayStation at an early stage of development is demonstrated. A lateral beam profile optimization was performed using the RayStation model and experimentally validated, highlighting the practical value and clinical readiness of the framework. In addition, dose rate visualization was achieved through custom scripting, enabling dose rate display within the TPS for physician review.^30^, ^31^, ^32^, ^33^, ^34^

## Materials and methods

### Proton radiation unit

The proton therapy system used in this study is the IBA ProteusONE (Louvain-La-Neuve, Belgium) single-gantry proton unit. It is equipped with a superconducting synchrocyclotron capable of accelerating protons up to 228 MeV for PBS delivery. In clinical mode, proton energies range from 70 to 226 MeV through the use of an energy degrader. However, the degrader substantially reduces the proton fluence reaching the nozzle. To achieve the maximum possible proton fluence for FLASH-RT applications, only the highest nominal energy of 228 MeV was utilized.^35^, ^36^, ^37^ To further enhance fluence, beamline transmission and optics were re-optimized to improve overall efficiency. Consequently, the UHDR beam requires separate commissioning and characterization from the clinical beam. At present, the UHDR beam is available only at the gantry 0° position, with additional tuning for other gantry angles ongoing. Nevertheless, establishing a beam model in the TPS enables preclinical study design, research planning, and preliminary dose-rate modeling while preparing for full-angle implementation. Therefore, all measurements and data reported in this work were acquired at gantry 0°.

The distal end of the nozzle can be equipped with various beam-modifying accessories. A small snout, providing a maximum circular field size of 12 cm in diameter at the isocenter, can be attached to the accessory drawer and used to mount apertures and range shifters. The snout position can be adjusted along the beam axis to vary the air gap as needed.

Two custom brass apertures were used in this study: one with a field size of 2.5 × 2.5 cm and another with a field size of 2.0 × 3.5 cm. The original accessory drawer includes 2 slots designed to hold a 4 cm-thick aperture and a 4 cm-thick range shifter. For 228 MeV beam energy, the aperture slot was repurposed to accommodate an increased aperture thickness of 6.5 cm. All apertures were manufactured by DotDecimal, Inc (.decimal, Sanford, Florida, USA). The FLASH beamline has the same field size as the clinical beam (20 × 24 cm). This work will focus on validating preclinical field sizes using apertures to achieve a well-defined penumbra.

### TPS commissioning data measurements

The TPS is RayStation, Version 11B (Raysearch Lab, Stockholm, Sweden). The data needed for beam commissioning are virtual source position, spot size in air at various planes, integrated depth dose (IDD), and absolute dose calibration.^38^

#### Virtual source position

A plan consisted of 4 spots at position [2.5 cm, 2.5 cm], [2.5 cm, −2.5 cm], [−2.5 cm, −2.5 cm], [−2.5 cm, 2.5 cm] were delivered in the air, and the measurements are taken along the z-axis (patient anterior-posterior (AP) direction), z = 0, 10, 20, 30, and 40 cm. The spot positions are measured using a 2D scintillation detectors (Lynx, IBA dosimetry, Schwarzenbruck, Germany), as shown in Figure 2A. Lynx saturates at high dose levels, and the measurements are done with a low dose (1 pulses per spot) at UHDR with the lynx iris closed at the minimum 30%. The virtual source distance was calculated using geometric fitting of the spot position at various planes.

#### Spot profile measurements

A single unscanned spot was delivered in air, and the measurements are taken along the z-axis (z = −5, 0, 10, 20, 30, and 40 cm) using Lynx. Again, the lynx is set at iris 30% with only 1 spot. The same spot is repeated 10 times for an average image for final analysis. The spot profiles in x- and y-directions are extracted from the Lynx spot image determined by the central maximum intensity. The profiles then were imported in RayStation commissioning module to determine the spot phase space divergence and correlation coefficients. The profiles at different planes are then fitted to the Fermi-Eyges transport equations:

$$\overset{®}{r^{2}}\left(z\right)=\overset{®}{r^{2}}\left(0\right)+2\overset{®}{r\theta }\;\left(0\right)\;z+\overset{®}{\theta^{2}}\left(0\right){\;z}^{2}$$

where z represents the positions the spot measurements, and $\overset{®}{r^{2}}$ is the spot size at isocenter, $\overset{®}{\theta^{2}}$ is the angular variance, and $\overset{®}{r\theta }$ is the spatial-angular covariance.

#### Integrated depth dose

The measurement of IDD in our facility is limited by the availability of the UHDR beam at gantry 0 only. We approached the acquisition of IDD in 4 practical steps. First, we acquired the IDD using the Giraffe multi-layer ionization chamber detector (IBA dosimetry, Schwarzenbruck, Germany) with no build up, 5 cm solid water, and 10 cm solid water. Second, the effective thickness of 5 cm solid water is determined by the R90 differences between IDD measured with 5 cm solid water and 10 cm solid water (WET = 5.17 cm). Third, the R90 of the UHDR beam is then determined to be the R90 value of the analyzed IDD data with 5 cm solid water added by 5.17 cm. Lastly, the fourth step, the 226 MeV clinical beam (R90 = 32.00 cm) is shifted to match the IDD for the UHDR beam from step 3, which is called the shifted UHDR IDD. The Giraffe measurement was performed using the UHDR beamline with reduced dose rate to the CONV level. At this range, the dose delivered is no longer accurate in respect to the requested MU, but the energy spectrum still holds. For a facility that has 3D scanning tank with Gantry 0 access, or later when Gantry 90 FLASH beam is commissioned, this step will be greatly simplified.

#### Absolute dose calibration

The absolute dose output was measured with a PPC05 parallel plate ionization chamber (PPC05, IBA dosimetry, Schwarzenbruck, Germany). PPC05 has a diameter of collecting electrode of 9.9 mm, and the electrometer is Dose-1 (IBA dosimetry, Schwarzenbruck, Germany). PPC05 is placed in solid water at 2 cm, and the surface of the solid water is set at the isocenter. The irradiation field is 3 x 3 cm square field with 5 mm spot spacing, resulting in 49 spots with UHDR beam line. Each spot carries 20 MU. For PPC05 measurements, the correction factor of Pion and Ppol was measured at UHDR dose rate, to be 1.0097 and 1.0042.^26^

#### Brass aperture as snout accessory

Snout supported block is also incorporated in the machine model. The aperture is made of brass material, and the thickness of the block is 6.5 cm with a milling accuracy of 5 mm drill bit. The block will move with the snout position verified with various air gaps.

### Flash delivery sequence

The IBA ProteusONE synchrocyclotron has a pulse repetition rate of 1 kHz and a fixed pulse width of 10 μs. The delivery dose rate is determined by Faraday cup placed at the isocenter, and the charges per pulse were measured and calculated for the average dose rate computation.

**Figure 1:**
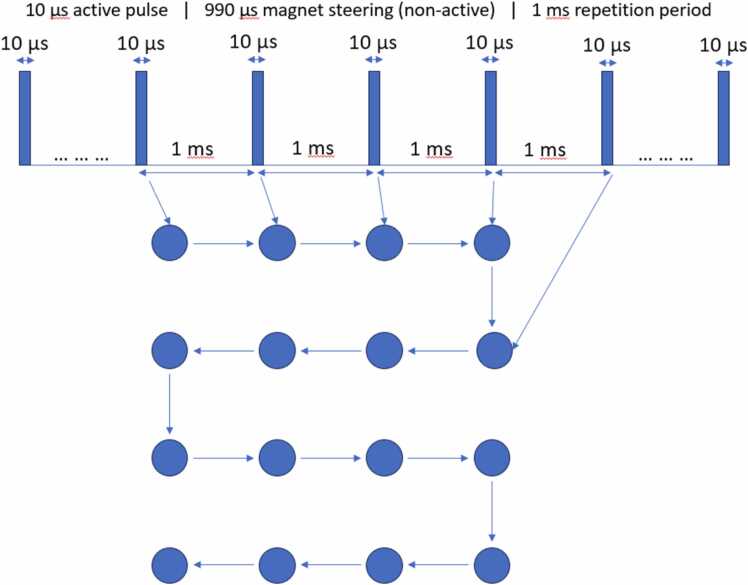
The diagram for the delivery sequence of IBA ProteusOne FLASH delivery. The dose was delivered completely to a given spot. The steering magnet adjusts the beam to the next targeted position during the non-active period.

This pulse structure is well-defined and governs both the temporal and spatial characteristics of FLASH dose delivery. As illustrated in Figure 1, the accelerator produces proton pulses every 1 ms, with each active pulse that delivers proton lasting for 10 μs. During this active period, the proton beam is directed toward the designated target spot position, corresponding to a single spot within the treatment field. There is a maximum MU that can be delivered in 1 pulse, approximately 5 MU. The desired dose will be completely delivered to a spot before moving to the next spot position. The position of this spot is indicated schematically as a blue dot in Figure 1. Between consecutive pulses, during the non-active interval (∼990 μs), no protons are extracted from the synchrocyclotron. This interval is utilized by the scanning magnet system to reposition the beam to the next planned lateral location within the target grid. Upon completion of the magnet transition, the next 10 μs pulse is delivered to that new location. This pulse-by-pulse spot delivery continues sequentially until the entire energy layer is completed. A caveat for this delivery scheme is that the maximum allowable spot-to-spot distance of approximately 8 mm is required. If the required positional change exceeds this limit, the system will intentionally skip 1 or more beam pulses to provide sufficient time for magnet repositioning before resuming delivery. All pulse events, including timing, position, proton charge, and potential delays, are continuously recorded in the machine delivery log files, which serve as the record for treatment verification and time-resolved analysis of delivery dynamics.

### Point dose validation

The point dose validation was performed with additional detectors that have smaller volumes. The summary of the detector used and cross-calibration procedure was listed in Table 1. The suitability of the detectors used for the UHDR beam was investigated previously, where the details on the cross-validation process can also be found in the literature.^26^, ^27^ For the 3 x 3 cm square irradiation field with 5 mm spot spacing, measurements at 4 depth (2, 4, 6, and 8 cm) in solid water is taken. At 2 cm solid water depth, measurements with 4 square irradiation field were taken (field size = 3, 2.5, 2, and 1.5 cm). Additional field sizes are measured with PPC05, field size = 10, 7, 5, and 4 cm. Measurements of 2 cm solid water depths were taken in the presence of apertures. Lastly, for the 3 x 3 cm field, additional measurements were taken when the solid water surface were set at 10 and 20 cm upstream of the isocenter.

**Table 1:** Summary of point radiation detectors used in the study.

Detector	Detector collection property	Calibration notes
PPC05	Collection Diameter: 9.9 mm Collection Thickness: 0.6 mm Volume: 46 mm^3^	ADCL calibrated for N_dw_, dose was calculated based on P_TP_, P_pol_ and P_ion_ correction from TRS398 formula.
Razor Chamber	Cylinder Diameter: 2.0 mm Cylinder Length: 3.6 mm Volume: 10 mm^3^	N_dw_ cross calibrated with PPC05 under conventional beam, dose was calculated based on P_TP_, P_pol_ and P_ion_ correction from TRS398 formula.
Nano Razor Chamber	Sphere Diameter: 2.0 mm Volume: 3 mm^3^	N_dw_ cross calibrated with PPC05 under conventional beam, dose was calculated based on P_TP_, P_pol_ and P_ion_ correction from TRS398 formula.
PTW MicoDiamond	Collection Diameter: 2.2 mm Collection Thickness: 0.001 mm Volume 0.004 mm^3^	N_dw_ cross calibrated with PPC05 under conventional beam.

### 2D dose validation

2D dose profile validations were taken using Lynx and EBT-XD films. The comparisons were performed with various fields at 2 cm depth, 4 open fields (field size = 3cm, 2.5 cm, 2 cm, and 1.5 cm), and 2 aperture fields at 6 cm air gaps.

The Lynx measurements were performed with the smallest Iris opening of 30%, and the plan has 5 MU per spot. The film is irradiated with the same plan but 40 MU per spot. The lynx intensity image is rescaled by point dose to convert the intensity image to dose image for comparison. This is performed in MyQA software.

The EBT-XD film for proton dosimetry was described previously.^25^, ^29^ Film calibration was performed for each lot with a uniform proton field of 10 x 10 cm, with an energy of 226 MeV.^39^ The film scanner used to digitize the film is an Epson scanner, model Expression 11000XL (Epson America, Inc, Los Alamitos, CA, USA). The scanning resolution is 300 dpi with no image processing or color correction applied. The resolution is consistent with all our previous work that requires high spatial resolution.^25^, ^40^ During analysis, the resolution was re-binned to 0.5 mm spatial resolution for noise reduction. Dose conversion was performed using the IBA MyQA film panel application within the MyQA framework (IBA Dosimetry, Schwarzenbruck, Germany).

The plane dose that is correspondent to the measurement plane was exported from RayStation and compared in MyQA patient analysis. Gamma analysis with the RayStation dose as the reference and measurements as the comparison dose distribution were performed using various gamma criteria (1 mm/1%, 1 mm/2%, 1.5 mm/1.5%, 2 mm/2%, and 3 mm/3%).

### PBS dose rate calculation via RayStation scripting

The definition of dose rate in proton PBS for FLASH applications has been widely discussed in recent literature.^3^, ^19^, ^30^, ^32^ However, no consensus has yet been reached regarding which dose rate metric best correlates with clinical outcomes or biological effectiveness. Therefore, it is recommended that multiple dose rate definitions be reported alongside conventional dose information during the early stages of FLASH research and planning. In this study, we incorporated 3 dose rate definitions from the literature to demonstrate the implementation and visualization of PBS dose rate metrics within the TPS.

#### PBS dose rate, as defined by Folkerts et al can be written as^32^

(1) $${\dot{D}(x)}^{\mathrm{PBS}}=\frac{D\left(x\right)-2D^{\mathrm{th}}}{t_{1,i}-t_{0,i}}$$

where $t_{1,i}=t_{i}(D_{i}-D^{\mathrm{th}})$ and $t_{0,i}=t_{i}(D^{\mathrm{th}})$. The denominator basically means the time that pixel experienced above the threshold dose $D^{\mathrm{th}}.\;$The threshold dose can be somewhat arbitrary, and in our study, 0.5 Gy is used as 2.5% of the prescription dose.

#### Max percentile dose rate

In this definition, the PBS dose rate is defined as a given percent of the dose at a point divided by the time interval between reaching 2.5% of the dose at the point. For a given pixel, there can be more than 1 time window the dose reaches the threshold. The maximum percentile dose rate^33^ extends the concept by including all the time windows where the accumulated dose reaches a certain dose threshold $D_{\mathrm{PERC}}$.

(2) $${\dot{D}(x)}^{\mathrm{MP}}=\max\frac{\int_{t_{0}}^{t_{1}}d_{i}(t)\mathrm{dt}}{t_{1}-t_{0}},\mathrm{where}\int_{t_{0}}^{t_{1}}d_{i}(t)\mathrm{dt}\geq D_{\mathrm{PERC}},t_{1}>t_{0}$$

The threshold dose $D_{\mathrm{PERC}}\;$can be defined in the code as input. In this work, DR50 means $D_{\mathrm{PERC}}$ set to be 50% of the prescription dose. This calculates the maximum dose rate to deliver a threshold dose out of the total dose delivered. In this definition, voxels receiving less than threshold will not be calculated.

#### Voxel-wise maximum instantaneous dose rate

For this definition,^30^ each voxel (that receives a minimum dose threshold, 0.5 Gy used for consistency) the dose $\Delta D_{i}$ is computed for each time interval Δt during the delivery but only store the maximum value $D_{i}$. The equation is expressed as:

(3) $${\dot{D}(x)}^{\mathrm{MVox}}=\frac{\max(\Delta D_{i})}{\Delta t}$$

In this work, we chose Δt to be 1 ms.

### 2D profile optimization for transmission FLASH pre-clinical experiments

In addition to the standard field validation, optimized 2D fields are also performed and evaluated. The UHDR beam spot profile is different from clinical beams, and thus, the same spot arrangement under the aperture yields different lateral profiles. This intrinsic difference made comparative studies between conventional dose rate and UHDR dose rate difficult. Without the beam model implemented in RayStation, lateral beam profile optimization can be performed experimentally by iteratively adjusting the spot weights, measuring the resulting profiles using the Lynx detector, and comparing them with the corresponding conventional dose profiles until satisfactory agreement is achieved. This tedious process can be improved with the RayStation model of the transmission UHDR beam model. Spot weights were adjusted manually in RayStation. The similarity of the lateral profiles at depths of 2 cm were evaluated using 2D gamma analysis.

## Results

### Faraday cup measurements

**Figure 2:**
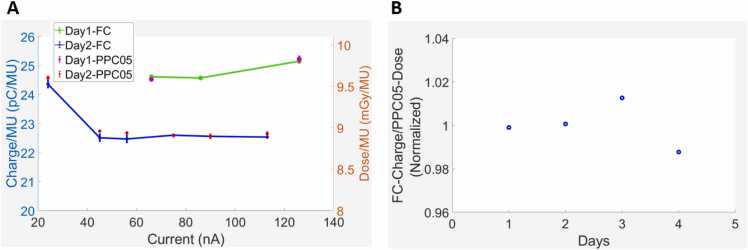
Faraday cup and PPC05 measurements on the UHDR beam linearity and consistency. (A) For the Faraday cup, the charge per MU is also plotted on the left axis, and for PPC05, the dose per MU is plotted on the right axis. (B) The ratio between the charge per MU with Faraday cup and the Dose per MU measured by PPC05 on multiple days.

The range of achievable beam current under the UHDR setting was measured using a Faraday cup, and the results are presented in Figure 2. The Faraday cup used in this study is a custom-built, vacuum-less design as previously described.^26^, ^41^ Figure 2A shows the charge per monitor unit (MU, left axis) ranging from 24 to 126 nA, measured on 2 separate days (spaced more than 30 days). The corresponding dose per MU (right axis) is also plotted.

Two key observations can be drawn from these data. First, the charge per MU remains consistent across the current range of 45 to 126 nA. Below 45 nA, however, the calibration curve deviates significantly, rendering the calibration factor used in RayStation invalid; therefore, 45 nA can be considered the lower operational limit for UHDR calibration. Second, the MU definition in the cyclotron’s ionization chamber can vary slightly between days due to the absence of temperature and pressure corrections. Despite this, the linearity of the charge–dose relationship remains within 2% throughout the 45 to 126 nA range.

If the ratio between the charge per MU (from the Faraday cup) and the dose per MU (measured by PPC05) is evaluated, the day-to-day consistency remains within 2%, as shown in Figure 2B. These results demonstrate that, although minor variations exist in the absolute dose calibration between days, the dose output is stable within the UHDR range of 45 to 126 nA. Such day-to-day differences can be easily corrected by performing a single daily Faraday cup measurement prior to each experiment. In the future, with a dedicated UHDR ionization chamber, dose control can be further refined and stabilized.

### RayStation model for ProteusOne transmission FLASH

In this section, we presented the RayStation model for ProteusOne transmission FLASH, including spot measurements, energy spectrum, and absolute output dosimetry. The measured data were fitted using the Monte Carlo auto-model after input.

#### Virtual source distance

**Figure 3:**
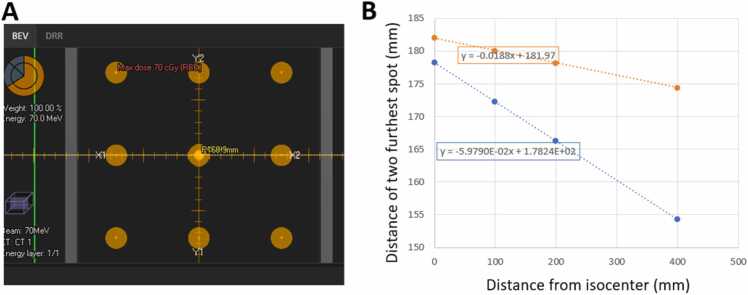
Measurement of the SAD. (A) The spot map used to measure SAD with Lynx. (B) The fitting of the measurements to obtain beam SAD, the blue curve is the Y direction, and the orange curve is the X direction.

At a given plane, the distances between spots are calculated separated for X and Y directions. Figure 3B is the plot of the gap versus the distances between spots measured at that spot. Based on the fitted linear curve, the virtual source distance is calculated to be 289 and 960 cm in X and Y directions. This is similar to what clinical beam line has.^29^

#### Spot profiles

**Figure 4:**
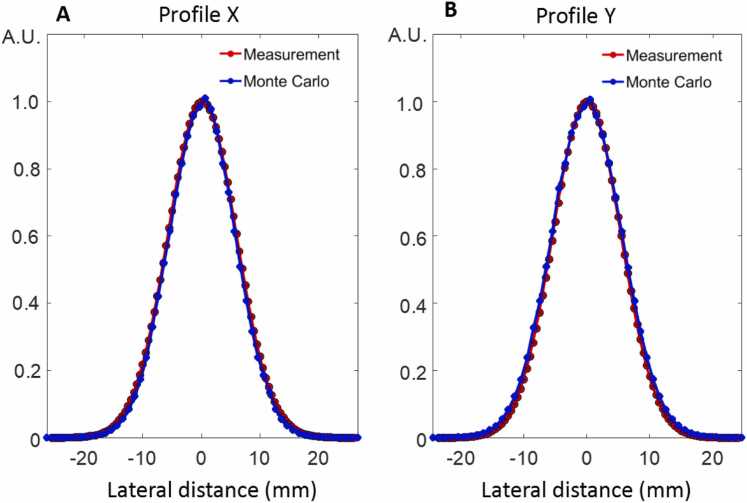
The plot of the spot profile in the X and Y directions at the isocenter. (A) the spot profile in the X direction, showing the measurement and the fitted Gaussian shape from the RayStation Monte Calo calculation, corresponding to spot sigma of 5.8 mm. (B) Similarly, the spot profile in the Y direction, corresponding to spot sigma of 5.4 mm.

The spot profiles at the isocenter and the Gaussian fit are shown in Figure 4. The fitted spot size (spot sigma) is 5.8 mm in X-Profile and 5.4 mm in Y-Profile. However, RayStation TPS treats the spot as symmetrical shape, and the spot size (spot sigma) in TPS is fitted to be 5.62 mm. The fitted Angular spread is 2.22 mRad.

#### Energy measurements

The TPS input is called the shifted UHDR IDD as described in the method session. We then constructed the Giraffe UHDR IDD by combing the first 10 cm of IDD measurement with no buildup and the rest of the IDD measurements with IDD measurement with 5 cm solid water shifted by 5.17 cm. Then the shifted IDD and Giraffe IDD is compared by the following parameters, R80, R90, and Falloff. The results are listed in Table 2.

**Table 2:** The IDD measurements for the UHDR beam.

	R80 (cm)	R90 (cm)	Falloff (cm)
Giraffe UHDR IDD	32.46	32.36	0.49
Shifted UHDR IDD	32.45	32.36	0.48

The difference is all within 0.5 mm, which is well within the TG224 tolerance for annual QA tolerance for clinical beam .^42^

#### Output dose calibration

The suitability of the PPC05 chamber for synchrocyclotron UHDR beam measurement has been demonstrated in Figure 2. The output calibration is calculated to be 0.163 Giga protons per MU. This is consistent over the range of UHDR from 45 to 126 nA, within 1% difference. The MU is scaled based on the daily FC and PPC05 measurements as described in Section “Faraday cup measurements.”

### Point dose validation

Comprehensive point dose validation was performed to evaluate the validity of the model. The results are shown in Table 3, Table 4, Table 5, as results are sorted in 4 sets for visual clarity. The RayStation dose values were considered of the actual detector volume in dose scoring, and thus the value can be slightly different for the same field with different detectors. Each experimental measurement was repeated 3 times or more, and the measurement standard deviation is also presented.

**Table 3:** Point dose validation for open fields with various field sizes.

Field size (cm)	PPC05			Razor chamber			nanoRazor chamber			microDiamond		
	Meas (Gy)	Ray (Gy)	%	Meas (Gy)	Ray (Gy)	%	Meas (Gy)	Ray (Gy)	%	Meas (Gy)	Ray (Gy)	%
1.5	7.90 ± 0.04	7.83	0.9	8.46 ± 0	8.27	2.3	8.43 ± 0.01	8.27	1.9	8.36 ± 0.02	8.26	1.2
2	9.05 ± 0.01	8.91	1.6	9.29 ± 0.01	9.19	1.0	9.27 ± 0.02	9.19	0.8	9.22 ± 0.02	9.23	−0.1
2.5	9.52 ± 0.01	9.41	1.2	9.60 ± 0.06	9.55	0.5	9.59 ± 0	9.55	0.4	9.52 ± 0.03	9.54	−0.2
3	9.63 ± 0.02	9.61	0.2	9.71 ± 0.03	9.72	−0.1	9.69 ± 0.13	9.72	−0.3	9.72 ± 0.01	9.72	0
4	9.71 ± 0.04	9.72	−0.1									
5	9.87 ± 0.03	9.76	1.1									
7	9.83 ± 0.03	9.76	0.7									
10	9.94 ± 0.01	9.82	1.2									

Meas, Measurements; Ray, RayStation.

**Table 4:** Point dose validation for 3 x 3 cm square irradiation fields with various field sizes and 2 different SSD.

Detector depth	PPC05			Razor chamber			nanoRazor chamber			microDiamond		
	Meas	Ray	%	Meas	Ray	%	Meas	Ray	%	Meas	Ray	%
2	9.63 ± 0.02	9.61	0.2	9.71 ± 0.03	9.72	−0.1	9.69 ± 0.13	9.72	−0.3	9.72 ± 0.01	9.72	0
4	9.66 ± 0.11	9.7	−0.4				9.73 ± 0.01	9.8	−0.7			
6	9.68 ± 0.13	9.78	−1.0	9.72 ± 0.03	9.82	−1.1	9.67 ± 0.09	9.82	−1.5	9.59 ± 0.02	9.80	−2.2
8	9.70 ± 0.03	9.78	−0.8				9.64 ± 0.11	9.82	−1.8			
Upstream of isocenter												
10	10.08 ± 0.15	10.05	0.3									
20	10.51 ± 0.12	10.51	0									

Meas, Measurements; Ray, RayStation.

**Table 5:** Point dose validation for aperture fields with 2 field sizes.

Field size	PPC05			Razor Chamber			nanoRazor Chamber			microDiamond		
	Meas	Ray	%	Meas	Ray	%	Meas	Ray	%	Meas	Ray	%
2.5 × 2.5	10.65 ± 0.07	10.47	1.8	10.77 ± 0.01	10.6	1.6	10.8 ± 0.01	10.6	1.9	10.64 ± 0.02	10.57	0.6
2 × 3.5	10.44 ± 0.04	10.33	1.0				10.6 ± 0.01	10.4	2.0			

Meas, Measurements; Ray, RayStation.

The first set of data is taken without aperture with various field sizes. Four square irradiation fields were taken (field size = 3, 2.5, 2, and 1.5 cm) at depth of 2 cm in solid water with several detectors, and addition field sizes were measured with PPC 05, field size = 10, 7, 5, and 4 cm. The results are shown in Table 3.

The second set of data is again taken without aperture with 3 x 3 cm square irradiation field, measured at 4 depths (2, 4, 6, and 8 cm) in solid water. The results are shown in Table 4. The third set of data is again taken without aperture for the 3 x 3 cm field at 2 cm depth in solid water by placing the solid water surface at 10 and 20 cm upstream of isocenter.

Lastly, additional measurements are taken with 2 brass apertures designed for small animal irradiation. The results are shown in Table 5.

### 2D dose validation

**Figure 5:**
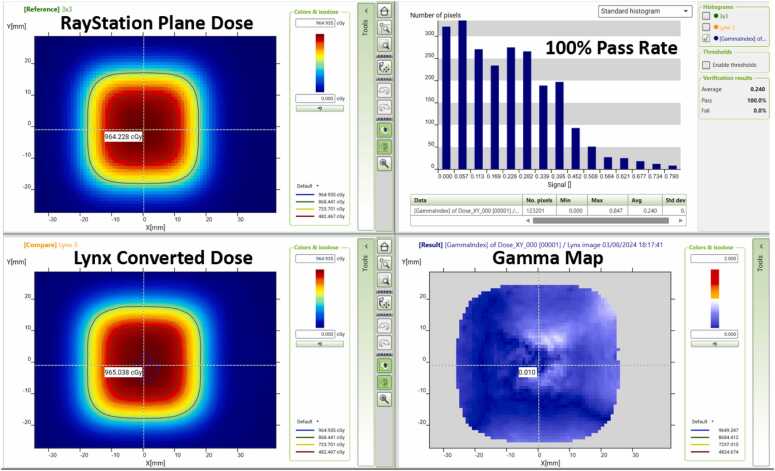
2D dose profile comparison of RayStation calculation and Lynx measurement for the 2.5 x 2.5 cm aperture field.

**Figure 6:**
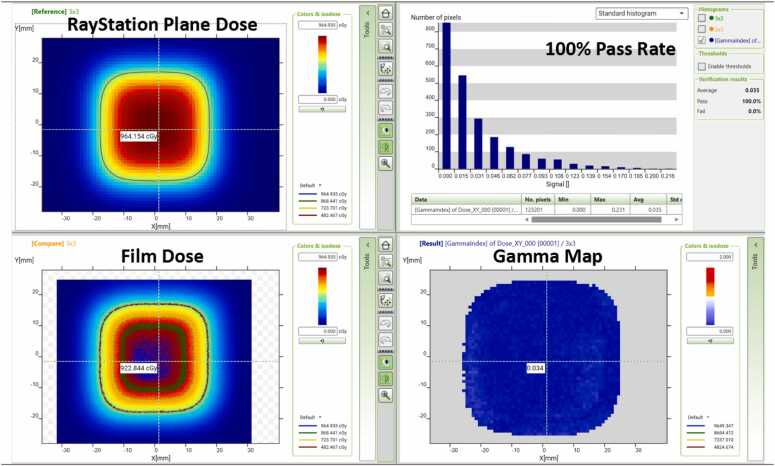
2D dose profile comparison of RayStation calculation and film measurement for the 2.5 x 2.5 cm aperture field.

The results for the 2D dose profile validation are shown in Figure 5, Figure 6, and Table 6. Table 6 included all the comprehensive gamma analysis for the open fields and 2 aperture fields at depths of 2 cm.

**Table 6:** Summary of the comprehensive gamma analysis of the open and aperture fields between RayStation calculations and Lynx and Film 2D profile measurements.

	Lynx converted dose			FILM		
Field size	Gamma analysis					
	2%/2 mm	2%/1 mm	1%/1 mm	2%/2 mm	2%/1 mm	1%/1 mm
3	100	96.5	93.3	100	100	100
2.5	100	98.5	97.5	100	100	99.7
2	100	98.6	97.2	100	96.4	93.5
1.5	100	98.3	95.5	100	99.8	99.4
2.5AP	100	95.3	95.3	100	100	99.9
2.0 × 3.5AP	100	93.8	90.6	100	99.9	99.5

AP, Aperture.

We presented the profile comparison and gamma analysis for the 2.5 x 2.5 cm aperture field using Lynx in Figure 5. Lynx measurements were scaled to convert the count to dose. The gamma analysis using the 2 mm/2% criteria shows 100% passing rate, indicating excellent agreement with the lateral profile prediction of the RayStation dose calculation. Similarly, the comparison between RayStation and film measurement for the 2.5 x 2.5 cm aperture field is shown in Figure 6. This approach will prepare us the workflow for 2D dose QA implementation in the future.

### Dose rate display in TPS

**Figure 7:**
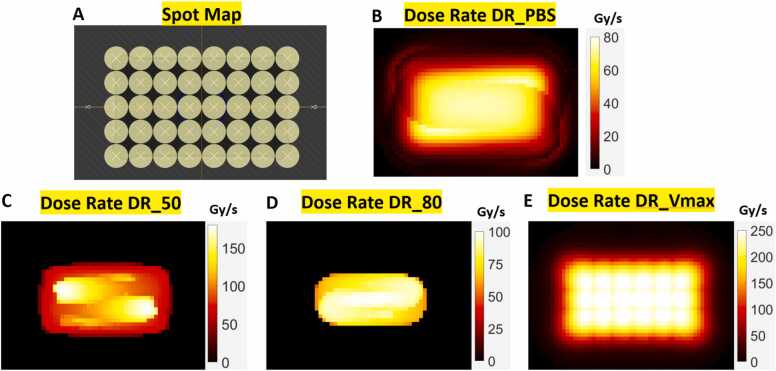
The dose rate profile using various dose rate definitions. (A) Uniform spot map used UHDR beam, with spot weight of 39.7 MU to deliver 20 Gy. (B) dose rate profile using the definition described in Section “PBS Dose Rate, as Defined By Folkerts et al Can Be Written As.” (C) and (D), dose rate profile using the definition described in Section “Max Percentile Dose Rate,” using 50 and 80 percent of Rx (DR_50 and DR_80). (E) dose rate profile using the definition described in Section “Voxel-Wise Maximum Instantaneous Dose Rate.”

For the 2.0 × 3.5 AP field, the dose rate calculation is performed, and the results are shown in Figure 7.

For the same spot map, the delivery sequence is described in Section “Flash Delivery Sequence.” The dose rate using various definition defined in Section “PBS Dose Rate Calculation Via Raystation Scripting” are shown. Notable differences are observed among the dose rate profiles derived from different definitions. The DR_PBS map (Figure 7B) shows a relatively uniform distribution across the field, reflecting the cumulative delivery characteristics of the PBS scanning pattern. In contrast, the percentile-based definitions (DR_50 and DR_80, Figure 7C and D) highlight the central high-dose region and yield a more confined high-dose-rate area as the percentile threshold increases. The DR_Vmax profile (Figure 7E) exhibits a distinct spot-like pattern that closely follows the scanning sequence, emphasizing instantaneous dose-rate variation across individual spots. Overall, the choice of dose-rate definition markedly influences both the magnitude and spatial pattern of the displayed dose rate, underscoring the importance of comprehensive definition when reporting FLASH dose rates.

### Dose profiles tuning for pre-clinical transmission FLASH experiment

**Figure 8:**
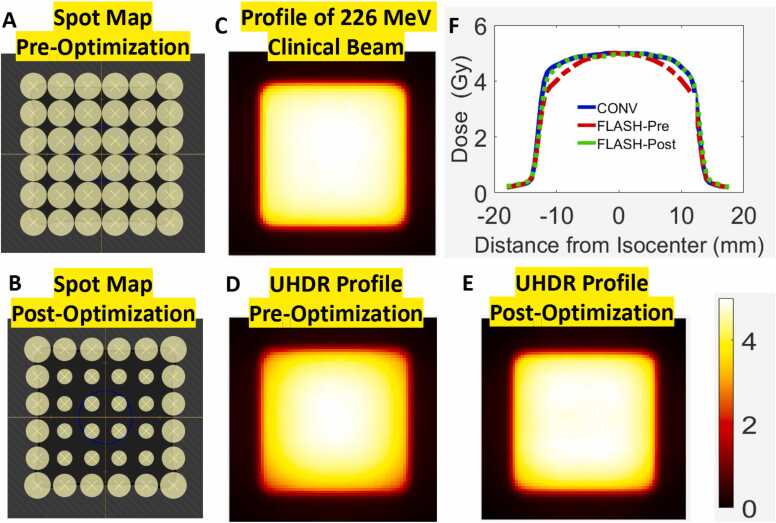
The measured profile by Lynx comparison of 226 MeV clinical beam and UHDR beam for 2.5 × 2.5 aperture field. (A) uniform spot map used by the 226 MeV clinical beam and UHDR beam (pre-optimization). (B) optimized spot map used UHDR beam (post-optimization). (C) Profile measured by Lynx of the 226 MeV clinical beam. (D) Profile measured by Lynx of the UHDR beam (pre-optimization). (E) Profile measured by Lynx of the 226 MeV clinical beam. (D) Profile measured by Lynx of the UHDR beam (post-optimization). (F) Profile across the center of the field showing the improvement of UHDR beam similarity post optimization to the 226 MeV clinical beam.

**Figure 9:**
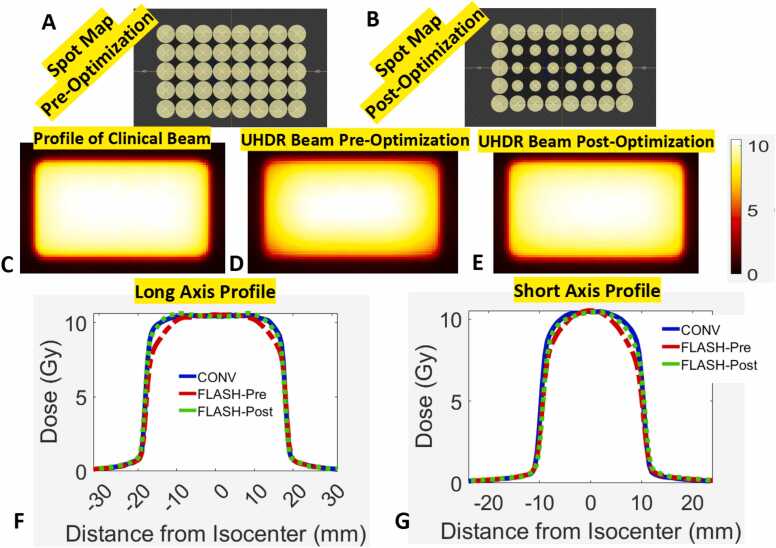
The measured profile by Lynx comparison of 226 MeV clinical beam and UHDR beam for 2.0 × 3.5 aperture field. (A) uniform spot map used by the 226 MeV clinical beam and UHDR beam (pre-optimization). (B) optimized spot map used UHDR beam (post-optimization). (C) Profile measured by Lynx of the 226 MeV clinical beam. (D) Profile measured by Lynx of the UHDR beam (pre-optimization). (E) Profile measured by Lynx of the 226 MeV clinical beam. (D) Profile measured by Lynx of the UHDR beam (post-optimization). (F) Profile across the center of the field in the long axis showing the improvement of UHDR beam similarity post optimization to the 226 MeV clinical beam. (G) Similarly, profile across the center of the field in the short axis showing the improvement of UHDR beam similarity post optimization to the 226 MeV clinical beam.

Profile optimization was demonstrated in Figure 8, Figure 9, and Table 7. The goal for the optimization is to adjust the spot weight of UHDR beam so that 2D dose profile of the UHDR and conventional beamline matches for pre-clinical FLASH experiment. Both Figure 8, Figure 9 are Lynx measurement comparison. Figure 6 shows the results for the 2.5 x 2.5 cm aperture field. If the same uniform spot map is used (spot map pre-optimization, Figure 8A), the profile uniformity between the 226 MeV clinical beam and the UHDR beam can be different. The difference is shown as the profile comparison in Figure 6F and the gamma analysis result of 85.9% and 52.5 % when 3 mm/3% and 2 mm/2% criteria are used, respectively. The optimization is performed in the RayStation manually to match the UHDR beam profile to the clinical beam profile, and the resulting spot map post-optimization is also shown in Figure 8B. The improvement is essential, as shown by the profile plot in Figure 8, and the gamma analysis result of 99.7% and 96.6% when 3 mm/3% and 2 mm/2% criteria are used, respectively.

**Table 7:** Gamma analysis shows the profile comparison between the 226 MeV clinical beam and the UHDR beam for both aperture fields.

	Pre-optimization		Post-optimization	
	3 mm 3%	2 mm 2%	2 mm 2%	1.5 mm 1.5%
2.5 × 2.5 Aperture	85.9	52.5	99.7	96.6
2.0 × 3.5 Aperture	86.0	62.4	99.7	95.9

Figure 9 shows the results for the 2.0 x 3.5 cm aperture field. The difference of the 226 MeV clinical beam and UHDR beam using uniform spot map (Figure 9A) is shown as the profile comparison in Figure 9F and G, and the gamma analysis result of 86.0% and 62.4% when 3 mm/3% and 2 mm/2% criteria are used, respectively. The optimization is performed in the RayStation manually to match the UHDR beam profile to the clinical beam profile, and the resulting spot map post optimization is also shown in Figure 8B. The improvement is essential, and the gamma analysis results of 99.7% and 95.0% when 3 mm/3% and 2 mm/2% criteria are used, respectively. These 2 apertures will be used for upcoming FLASH pre-clinical studies, and the reporting of the detailed physical dosimetry parameters are listed here for future references.

## Discussion

As an important step for translating the UHDR beam into clinical use for FLASH-RT applications, the ability to commission and characterize the beam in clinical platform can be essential. Charyyev et al has shown the commissioning process of the UHDR beam produced by the Varian ProBeam PBS proton system, with limited validation of the model.^37^ Yang et al also described the commissioning of the same system with more detailed measurements and validation on the dose-rate property of the system.^36^ Similarly, the IBA isochronous cyclotron UHDR beam has been shown to be incorporated in the TPS.^35^, ^43^, ^44^, ^45^ Our study focused extensively on the dosimetric validation of the beam model in TPS and demonstrated the excellent dose calculation accuracy with extensive point dose validations, 2D lateral profile validations, as well as demonstrating the valuable use of the commissioned beam model to facilitate comparative study.

The profile matching shown in our study is a unique need for IBA synchrocyclotron-based system. Most of the proton FLASH study are performed using isochronous cyclotron system, where the UHDR beam can reach low-current delivery at conventional dose rate.^46^ In contrast, the S2C2 system struggles to produce low current beams at the UHDR beam line setting. Therefore, the beam spot property used for UHDR FLASH study is different from the highest energy clinical beam used in control studies with conventional dose rate delivery. When performing biological FLASH experiment, the subject being tested needed to be irradiated under the same profile condition except the dose rate to demonstrate meaningful OAR protection under the FLASH beam. As a result, profile matching is essential for the S2C2 system when performing FLASH study and the beam model availability in TPS facilitates and speed up this process tremendously.

There have been several limitations of our study. First, the beam is only available at gantry zero currently. While the system has constantly been developed to include all beam angles, the beam property measured at gantry zero will give us the opportunity to establish the beam model in TPS. When the system is fully commissioned for all gantry angles, additional commissioning and QA process to validate the gantry angle consistency will be performed.^38^, ^42^ The list includes the consistency of spot size and shape, output, lateral profile symmetry, and flatness at various gantry angles. Second, the snout ionization chamber is not available for UHDR use now, and the charge count is relying on the cyclotron ionization chamber that was not meant for accurate dose counting.^47^ Therefore, we designed the daily Faraday cup charge measurement and PPC05 dose measurement of a reference field to scale the dose on the day of the experiment, as addressed in Section “Faraday Cup Measurements.” Third, the energy characterization was not performed using water tank. BluePhantom PT is the clinical 1D water tank that is available to us, and this is also a commonly used water phantom for IDD measurement by many proton facilities. The limitation of BluePhantom PT is the measurements can only be taken at Gantry 90°. We approximated the IDD based on Giraffe measurement in this study. Soon, when the UHDR beam is fully commissioned for all gantry angles, IDD measurements in water can be repeated for the refined beam model. Last but not least, only commonly used clinical detectors are used in this study, which does not provide accurate dose rate measurement due to the lack of temporal information.^17^, ^30^, ^48^, ^49^ However, this is an active area of research and beyond the scope of this study. In addition, the defined delivery structure in combination with log file record provides basic information for dose rate calculation, which was implemented in this work. This method is reliable given the highly consistent and predictable delivery structure of the IBA S2C2 system, allowing dose-rate metrics to be derived accurately from machine-reported timing and spot-delivery parameters. Overall, we were able to overcome all the obstacles and obtained the UHDR beam model in the RayStation platform, and the beam model is comprehensively validated for the depths and field sizes that are relevant for pre-clinical studies.

The current UHDR model is commissioned only for open beam with aperture abilities. In another word, the current planning ability is only for the transmission FLASH treatment modality. Particle FLASH technology started with transmission delivery technique, and in fact, this transmission approach was also the foundation of the first proton FLASH clinical trials.^22^, ^23^ However, the limitation of transmission proton FLASH is also obvious. The use of transmission FLASH diminishes the ability to achieve the zero dose beyond the Bragg peak inherent for particle therapy. As a result, transmission FLASH has demonstrated the loss of conformality compared to conventional proton plans. Alternatively, a promising strategy to use spread-out Bragg peak (SOBP) FLASH, which can be achieved with patient-specific range modulators to generate SOBP using a single energy layer FLASH beam. Several previous studies have shown superior OAR sparing and conformality using SOBP FLASH compared to transmission beam FLASH.^14^, ^45^, ^50^, ^51^, ^52^ The inclusion of vendor provided FLASH nozzle is beyond the scope of this study, as it is not yet available for IBA ProteusOne. Our work is also the first step toward the commissioning of SOBP FLASH.

The 2D profile validation presented in this work can be extended beyond UHDR commissioning. 2D ionization arrays are commonly used for patient specific QA, for instance, the IBA MatriXX series, and the PTW Octavius series. These devices usually have spatial resolution in the order of 2.5 to 7 mm, with electronics that are not suitable for UHDR beam. The use of Lynx is shown to be a viable method for FLASH lateral dose validation. When properly calibrated, Lynx can also be used to perform patient QA for small fields or highly modulated field in clinical beam. The use of Lynx provided quick 2D dose validation compared to EBT-XD film and can be included as a suitable UHDR QA in the future.

## Conclusion

In this work, we have shown the commissioning of the UHDR FLASH proton beam produced by the IBA S2C2 accelerator. The beam model is established in the RayStation TPS platform, and the dose calculation accuracy is validated extensively in our work.

## Data Availability

The dosimetry data presented in this study are available on request from the corresponding author; the patient data are not available to share due to patient privacy.

## CRediT authorship contribution statement

Yuting Lin: Conceptualization, Methodology, Investigation, Data curation, Formal analysis, Visualization, Software, Writing – original draft. Jufri Setianegara: Investigation, Software, Visualization, Writing – original draft. Aoxiang Wang: Investigation, Data curation. Nicolas Gerard: Investigation, Resources, Data curation. Jarrick Nys: Investigation, Resources, Data curation. Rudi Labarbe: Methodology, Validation, Writing – review & editing. Erik Traneus: Methodology, Software, Validation. Hao Gao: Conceptualization, Funding acquisition, Supervision, Writing – review & editing. Ronald C. Chen: Resources, Writing – review & editing.

## Declaration of Conflicts of Interest

The authors declare the following financial interests/personal relationships, which may be considered as potential competing interests: Hao Gao reports financial support was provided by the National Institutes of Health. If there are other authors, they declare that they have no known competing financial interests or personal relationships that could have appeared to influence the work reported in this paper.
